# A Lymph Node Staging System for Gastric Cancer: A Hybrid Type Based on Topographic and Numeric Systems

**DOI:** 10.1371/journal.pone.0149555

**Published:** 2016-03-11

**Authors:** Yoon Young Choi, Ji Yeong An, Hitoshi Katai, Yasuyuki Seto, Takeo Fukagawa, Yasuhiro Okumura, Dong Wook Kim, Hyoung-Il Kim, Jae-Ho Cheong, Woo Jin Hyung, Sung Hoon Noh

**Affiliations:** 1 Department of Surgery, Yonsei University Health System, Yonsei University College of Medicine, Seoul, Republic of Korea; 2 Brain Korea 21 PLUS Project for Medical Science, Yonsei University Health System, Yonsei University College of Medicine, Seoul, Republic of Korea; 3 Biostatistics Collaboration Unit, Yonsei University Health System, Yonsei University College of Medicine, Seoul, Republic of Korea; 4 Gastric Surgery Division, National Cancer Center Hospital, Tokyo, Japan; 5 Department of Gastrointestinal Surgery, University of Tokyo, Tokyo, Japan; 6 Department of Surgery, Samsung Medical Center, Sungkyunkwan University School of Medicine, Seoul, 06351, Korea; Queen Mary Hospital, HONG KONG

## Abstract

Although changing a lymph node staging system from an anatomically based system to a numerically based system in gastric cancer offers better prognostic performance, several problems can arise: it does not offer information on the anatomical extent of disease and cannot represent the extent of lymph node dissection. The purpose of this study was to discover an alternative lymph node staging system for gastric cancer. Data from 6025 patients who underwent gastrectomy for primary gastric cancer between January 2000 and December 2010 were reviewed. The lymph node groups were reclassified into lesser-curvature, greater-curvature, and extra-perigastric groups. Presence of any metastatic lymph node in one group was considered positive. Lymph node groups were further stratified into four (new N0–new N3) according to the number of positive lymph node groups. Survival outcomes with this new N staging were compared with those of the current TNM system. For validation, two centers in Japan (large center, n = 3443; medium center, n = 560) were invited. Even among the same pN stages, the more advanced new N stage showed worse prognosis, indicating that the anatomical extent of metastatic lymph nodes is important. The prognostic performance of the new staging system was as good as that of the current TNM system for overall advanced gastric cancer as well as lymph node—positive gastric cancer (Harrell C-index was 0.799, 0.726, and 0.703 in current TNM and 0.799, 0.727, and 0.703 in new TNM stage). Validation sets supported these outcomes. The new N staging system demonstrated prognostic performance equal to that of the current TNM system and could thus be used as an alternative.

## Introduction

In the field of gastric cancer, the fifth most common cancer and a major leading cause of cancer-related deaths worldwide [1] and particularly in East Asia [2,3], the application of appropriate staging systems has been a widely discussed issue in both Eastern and Western countries. The current staging system for gastric cancer is based on the extent of the primary tumor, the extent of lymph node (LN) metastasis, and the presence of distant metastases [4]. Although the staging for the extent of the primary tumor (T stage) is based on the depth of tumor invasion into the gastric wall, the staging for the extent of LN metastasis (N stage) has been converted from an anatomical location—based system to a numeric-based system [5,6]. Moreover, within this numeric-based system, the cutoff value of number of metastatic LNs defining the pN category has been changed. The purpose of this conversion was to predict prognosis more accurately [7–9] and to more easily perform comparisons with previous anatomical-based classifications [10]. However, the numeric-based N staging system has limitations, including its lack of information on the anatomical extent of the disease and its discordance between preoperative and postoperative N staging [11], as there is no way to determine the number of metastatic LNs prior to an operation; furthermore, the system cannot represent the extent of LN dissection despite the use of radical LN dissection (D2) as standard treatment [6,12,13].

The stomach is an organ to which blood is supplied by five main vessels (right and left gastroepiploic arteries, right and left gastric arteries, and short gastric artery); thus, it has an abundant and complicated lymphatic network system[14]. This complexity of the lymphatic network system for gastric cancer hinders the use of an anatomical-based system. However, the anatomical location of metastatic LNs is nevertheless important, as their locations depend on the location and severity of the primary cancer in the stomach; thus, it must be considered when staging gastric cancer. Therefore, an alternative N staging system that can simply and specifically represent the anatomic extent of the disease and provide accurate prognosis must be developed. To this end, we reclassified the LNs near the stomach and proposed a new staging system for gastric cancer based on a new N category.

## Methods

### Study design and participants

The data from patients who underwent gastrectomy for primary gastric cancer at Yonsei University Hospital between January 2000 and December 2010 were reviewed. The Institutional Review Board of Yonsei University Hospital agreed to exempt written informed consent from the participants and approved this study (4-2012-0798). To validate the new staging system, two hospitals in Japan were invited to participate in this study: one was the largest cancer center in Japan, National Cancer Center (NCC) Hospital (January 2000 to December 2007), and the other was a medium volume center, Tokyo University Hospital (TU; January 2004 to December 2010).

### Inclusion and exclusion criteria

All patients were pathologically confirmed to have primary gastric cancer. Minimally invasive surgery, such as laparoscopic or robotic gastrectomy, was excluded, and patients with any distant metastases (including peritoneal seeding and para-aortic LN metastasis) were excluded. Additional exclusion criteria were as follows: 1) cases in which the locations of LNs were not divided, 2) patients who underwent preoperative chemotherapy, and 3) patients with metastatic LNs of unclear locations. Ultimately, a total of 6025 patients were enrolled in this study and analyzed. In the validation sets, the criteria were identical for the NCC set (n = 3443), and minimally invasive surgery was additionally included in the TU set (n = 560).

### The extent of LN dissection and postoperative treatment

The surgical extent of LN dissection was based on the Japanese Classification of Gastric Carcinoma [10]. The operator divided the location of each LN just after the operation, according to the Japanese classification [6,10,15], and the pathologist reviewed and reported the status of each LN. Adjuvant therapy was administered when a patient was diagnosed with at least stage II gastric cancer, and the standard strategy of chemotherapy involved a 5-fluorouracil based regimen.

### Classification of LN groups based on anatomical locations

**Fig 1:**
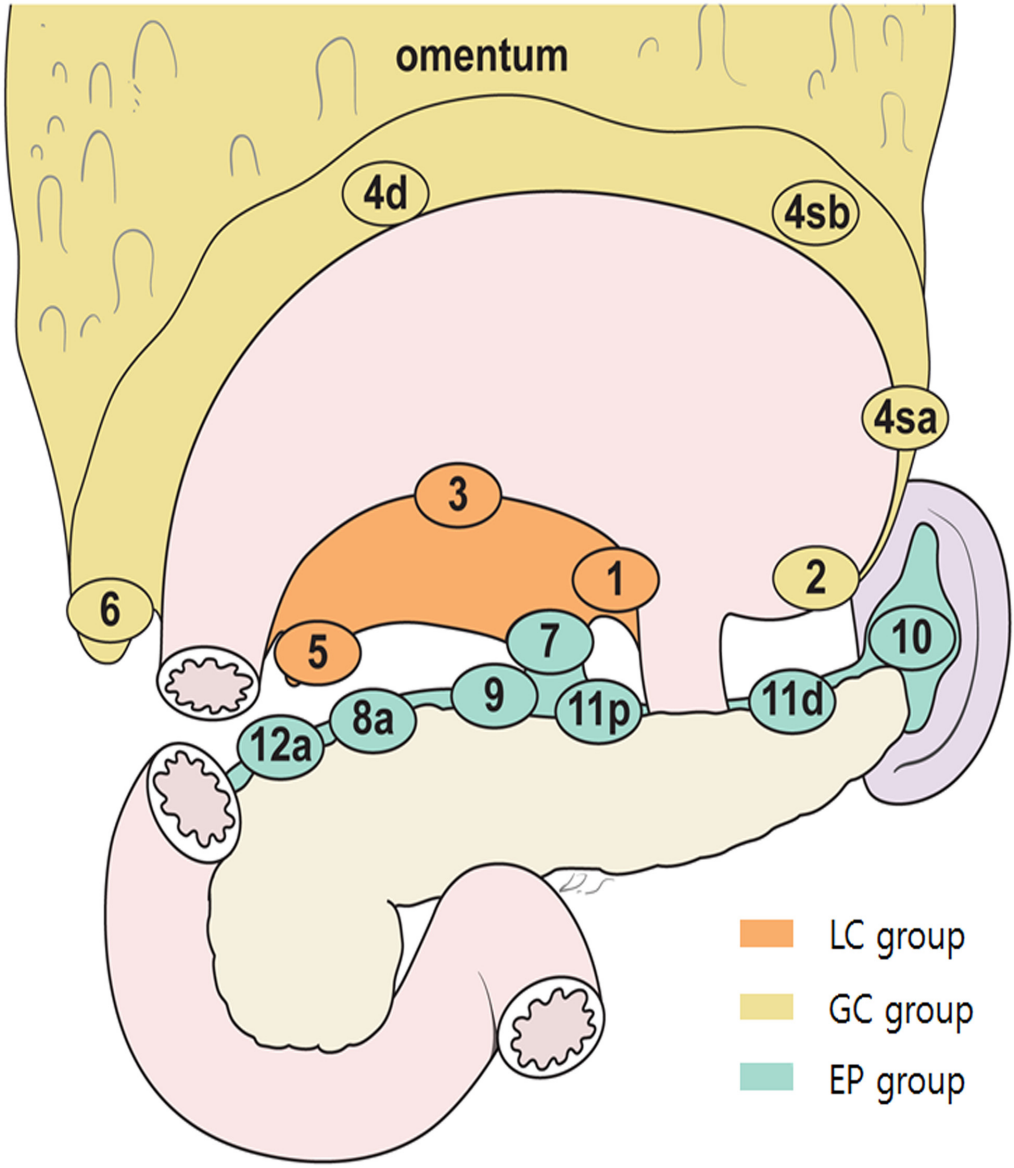
Classification of lymph node groups based on anatomical location. Lesser curvature (LC) group (station number 1, 3, and 5, according to Japanese classification), greater curvature (GC) group (station number 2, 4sa, 4sb, 4d, 6), and extra-perigastric (EP) group.

LN groups could be divided roughly into perigastric LN groups and extra-perigastric (EP) LN groups. Given the anatomical characteristics of the stomach, with lesser curvature (LC) and greater curvature (GC) sides, we divided perigastric LNs into two groups: LC (1, 3, 5) and GC (2, 4sa, 4sb, 4d, 6, and greater omentum). The remaining LNs, which were located in EP area (except the para-aortic area) were classified as the EP group (Fig 1). Regardless of the number of metastatic LNs, if any metastatic LN was involved in one group, we considered the corresponding group to be positive for metastasis. Finally, we stratified the status of LN groups into four categories: 1) new N0, indicated by no metastatic LN in any group; 2) new N1, indicated by one positive LN among three groups (positive LC alone, positive GC alone, or positive EP alone), regardless of number; 3) new N2, indicated by two positives out of three groups (positive LC + GC, positive LC + EP, or positive GC + EP), regardless of number; and 4) new N3, defined as positive results for all three groups (LC + GC + EP).

### A proposed new classification for the N staging system

To determine whether this new N classification could act as an N staging system, we compared prognostic performances with those of the current pN staging system, the 7^th^ edition of tumor-node-metastasis (TNM) from UICC [16]. The new N classification was combined with the current pT staging system, and a new TNM staging system was established. The new staging system was compared with the results of the current TNM system, including substages.

### Statistical analysis

A Kaplan-Meier analysis was performed to evaluate overall survival (OS). Survival data was represented as patient mean survival as it was not always possible to calculate median survival. The log-rank test and Cox proportional hazards model were used to compare the OS of the new staging system and the current TNM staging system. Kappa values were applied to evaluate the degree of conformity between the two staging systems. To compare the prognostic performance of each staging system, the Harrell C-index [17] (measuring the predictive accuracy of survival outcome) was used. A C-index of 1.0 indicated 100% predictive accuracy. In all cases, a *p*-value of less than 0.05 was considered to be statistically significant. The statistical analyses were performed using IBM SPSS 20.0 software (SPSS Inc., Chicago, IL, USA) and R software version 2.15.2 with the “survival” package.

## Results

### Patient demographics

The mean age of enrolled patients was 57.6 ± 11.8 years, and 33.6% were female (Table 1). Of 6025 patients, 1387 (23.0%) underwent total gastrectomy, and the mean number of retrieved LNs was 40.7 ± 15.5. The distribution of pT stage was similar to recently published reports [18–20]. The mean number of metastatic LNs was 3.2 ± 7.4 (8.5 ± 10.1 in lymph node—positive patients), and the median follow-up was 60 months.

**Table 1:** Patient demographics and characteristics of gastric cancer.

Variables	Yonsei (n = 6025)	NCC (n = 3443)	TU (n = 560)
**Age** (years), mean ± SD	57.6 ± 11.8	62.0 ± 11.6	65.6 ± 11.6
**Sex**			
Male, n (%)	4002 (66.4%)	2346 (68.1%)	395 (70.5%)
Female, n (%)	2023 (33.6%)	1097 (31.9%)	165 (29.5%)
**Extent of gastrectomy**			
Distal, n (%)	4633 (76.9%)	1649 (47.9%)	230 (41.1%)
Total, n (%)	1387 (23.0%)	809 (23.5%)	137 (24.4%)
^†^Others, n (%)	5 (0.1%)	985 (28.6%)	193 (34.5%)
**pT stage**			
mucosa, n (%)	1641 (27.2%)	946(27.5%)	123 (22%)
submucosa, n (%)	1369 (22.7%)	1169(34.0%)	207 (37.0%)
proper muscle, n (%)	780 (12.9%)	378(11.0%)	59 (10.5%)
subserosa, n (%)	665 (11.0%)	455(13.2%)	92 (16.4%)
serosa, n (%)	1506 (25.0%)	464(13.4%)	75 (13.4%)
adjacent organ, n (%)	64 (1.1%)	31(0.9%)	3 (0.5%)
**Number of metastatic LNs**			
Mean ± SD	3.2 ± 7.4 (8.5 ± 10.1)*	1.7±4.1(5.2±5.9)*	2.2 ± 6.1 (7.0 ± 9.3)*
0, n (%)	3794 (63.0%)	2326(67.6%)	386 (68.9%)
1–2, n (%)	716 (11.9%)	488(14.2%)	64 (11.4%)
3–6, n (%)	600 (10.0%)	335(9.7%)	51 (9.1%)
7–15, n (%)	541 (9.0%)	219(6.4%)	42 (7.5%)
≥16, n (%)	374 (6.2%)	75(2.2%)	17 (3.0%)
**Retrieved LNs**, mean ± SD	40.7 ± 15.5	43.0±72.9	38.4 ± 20.1

LN, lymph node; SD, standard deviation; NCC, National Cancer Center Hospital in Japan; TU, Tokyo University Hospital in Japan

*mean and SD of the number of metastatic LNs except pN0

^**†**^ pylorus-preserving gastrectomy or proximal gastrectomy

In validation sets, baseline characteristics were similar to the original set, except for the extent of gastrectomy: pylorus-preserving or proximal gastrectomy cases were more frequently performed in Japan. The median follow-up period was 77 months in the NCC set and 48 months in the TU set.

### Characteristics of the new N classification

**Fig 2:**
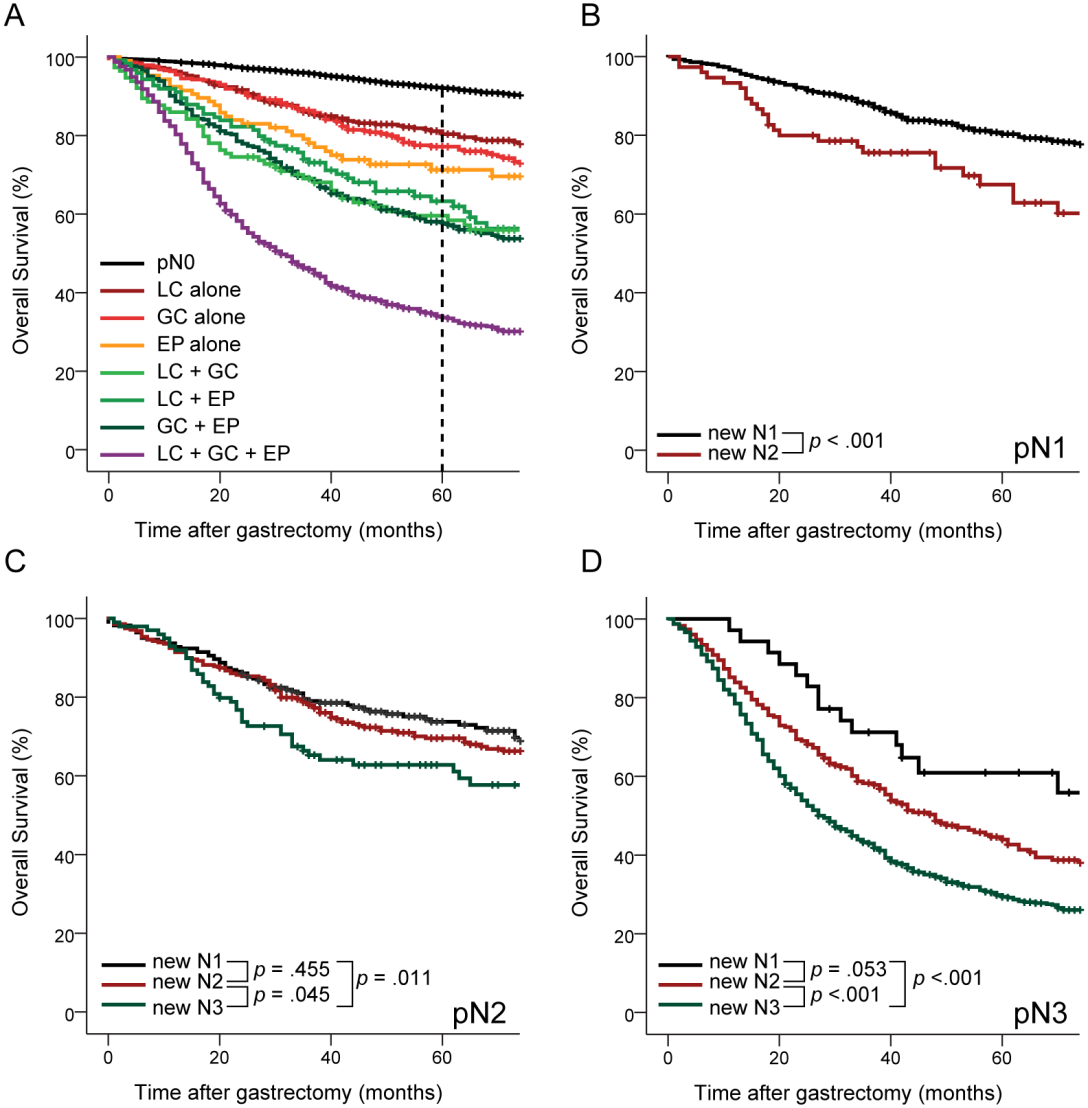
Overall survival for new N stages. A) patient survival among each combination of status-based lymph node groups, B) patient survival for each new N stage at pN1 (number of metastatic lymph nodes: 1–2), C) patient survival for each new N stage at pN2 (number of metastatic lymph nodes: 3–6), D) patient survival for each new N new stage at pN3 (number of metastatic lymph nodes ≥ 7). LC, lesser-curvature group; GC, greater-curvature group; EP; extra-perigastric group.

If the metastatic LN numbers were limited to only those near the perigastric area, the mean number of metastatic LNs was 3.6 ± 3.6, and if this area was expanded to the EP area, the mean number was 13.7 ± 11.9 (Table 2). The mean numbers of metastatic LNs according to the new N classification were 2.2 ± 1.9, 6.7 ± 5.2, and 17.5 ± 12.2 in the new N1, new N2, and new N3 groups, respectively. The Kaplan-Meier curves of OS for each combination of the status-based LN groups showed that the new classification provided a well-stratified prognosis for gastric cancer (Fig 2A).

**Table 2:** Number of metastatic lymph nodes for each combination of the three status-based groups.

	Number of patients (%)	Mean metastatic LNs ± SD
**Perigastric LN metastasis**	1137 (18.9%)	3.6 ± 3.6
**Extra-perigastric LN metastasis**	1094 (18.1%)	13.7 ± 11.9
**new N0**	3794 (63.0%)	0
**new N1**	898 (14.9%)	2.2 ± 1.9
LC alone	407 (6.8%)	2.2 ± 1.8
GC alone	385 (6.4%)	2.3 ± 2.0
EP alone	106 (1.8%)	2.1 ± 1.8
**new N2**	583 (9.7%)	6.7 ± 5.2
LC + GC	345 (5.7%)	6.5 ± 4.8
LC + EP	124 (2.1%)	6.5 ± 4.8
GC + EP	114 (1.9%)	7.5 ± 6.4
**new N3**	750 (12.4%)	17.5 ± 12.2
LC + GC + EP	750 (12.4%)	17.5 ± 12.2

LN, lymph node; SD, standard deviation; LC, lesser-curvature group; GC, greater-curvature group; EP, extra-perigastric group;

### Comparison of survival among new N stages for each pN stage

The differences in patient survival among the new N stages according to pN stage (according to 7^th^ edition of the TNM [16]) are presented in Fig 2B, 2C, and 2D. When the numbers of metastatic LNs were one or two (pN1), the new N2 group had a poorer prognosis than the new N1 group (Fig 2b) with statistical significance (*p <* 0.001). When the number of metastatic LNs was between 3 and 6 (pN2), there was no significant difference between the new N1 and new N2 groups; however, the prognosis of the new N3 group was worse than the new N2 group and the new N1 group (Fig 2c; *p* = 0.045 and 0.011, respectively). For pN3 (metastatic LNs ≥ 7), the prognosis of the new N3 group was worse than the new N1 and new N2 groups (Fig 2d; *p* < 0.001 for both).

### Relationship between the new staging system and the TNM 7^th^ edition system

The patient distributions of the new N classification and the pN staging system of the TNM 7^th^ edition are shown in Table 3. The kappa value between the pN stage of the TNM 7^th^ edition and new N classification systems was 0.803. In comparing the distributions of the new TNM staging system (combined pT stage of TNM 7^th^ edition with new N classification) and TNM 7^th^ edition system, the kappa value was found to be 0.856. The number of patients in each stage was well-distributed in both staging systems.

**Table 3:** Patient distributions for the TNM 7^th^ edition and new staging system.

	**TNM 7**^**th**^ **edition**								
		**pN0**	**pN1**	**pN2**	**pN3**	**total**			
	**new N0**	3794	0	0	0	3794			
	**new N1**	0	641	222	35	898			
	**new N2**	0	75	279	229	583			
	**new N3**	0	0	99	651	750			
	**total**	3794	716	600	915	6025			
**New staging system**		**Ia**	**Ib**	**IIa**	**IIb**	**IIIa**	**IIIb**	**IIIc**	**total**
	**new Ia**	2721	0	0	0	0	0	0	2721
	**new Ib**	0	641	31	0	0	0	0	672
	**new IIa**	0	23	433	54	6	0	0	516
	**new IIb**	0	0	19	513	74	13	0	619
	**new IIIa**	0	0	0	29	289	136	15	469
	**new IIIb**	0	0	0	0	42	237	144	423
	**new IIIc**	0	0	0	0	0	59	546	605
	**total**	2721	664	483	596	411	445	705	6025

kappa value = 0.803 (between new N stage and pN 7^th^ stage), 0.856 (between new TNM stage and TNM 7^th^ stage)

### Comparison of prognostic performance between the new staging system and the TNM 7^th^ edition system

The hazard ratios (HRs) for pN1, pN2, and pN3, compared to pN0, were 2.5, 3.8, and 11.0, respectively, in the current TNM 7^th^ edition. In the new N classification, the HRs of new N1, N2, and N3, compared to new N0, were 2.6, 5.3, and 11.2, respectively, which indicates that the new N stage system shows a similar distribution to the current N staging system (Table 4).

**Table 4:** Hazard ratios from Cox proportional hazard model and mean survival rates for the TNM 7^th^ edition and the new staging system.

		TNM 7th			New staging system		
		HR	*p*-value	mean survival (months)	HR	*p*-value	mean survival (months)
	**N0**	1	-	135.7 (133.3–138.1)	1	-	135.7 (133.3–138.1)
	**N1**	2.5 (2.1–3.0)	< .001	109.9 (105.8–113.9)	2.6 (2.2–3.1)	< .001	111.7 (107.7–115.6)
	**N2**	3.8 (3.2–4.4)	< .001	101.7 (95.4–107.9)	5.3 (4.6–6.2)	< .001	89.4 (83.6–95.2)
	**N3**	11.0 (9.7–12.4)	< .001	55.4 (51.8–59.1)	11.2 (9.9–12.8)	< .001	54.5 (50.4–58.5)
	**Ia**	1	-	143.3 (141.1–145.5)	1	-	143.3 (141.1–145.5)
**Yonsei**	**Ib**	2.1 (1.6–2.6)	< .001	128.1 (123.7–132.4)	1.9 (1.5–2.5)	< .001	129.2 (124.9–133.5)
	**IIa**	2.3 (1.8–3.0)	< .001	120.7 (116.1–125.2)	2.9 (2.3–3.6)	< .001	116.4 (111.7–121.0)
	**IIb**	4.5 (3.7–5.4)	< .001	107.6 (102.9–112.3)	4.1 (3.4–5.1)	< .001	109.9 (105.4–114.4)
	**IIIa**	5.5 (4.5–6.8)	< .001	98.4 (92.8–104.1)	6.4 (5.3–7.8)	< .001	95.0 (89.2–100.7)
	**IIIb**	9.3 (7.7–11.2)	< .001	82.3 (75.6–88.9)	11.0 (9.1–13.2)	< .001	76.2 (69.7–82.7)
	**IIIc**	19.3 (16.5–22.7)	< .001	49.3 (45.4–53.2)	20.2 (17.1–23.8)	< .001	47.6 (43.5–51.7)
	**Ia**	1		148.6(145.1–152.2)	1		148.6(145.1–152.2)
	**Ib**	1.4(0.9–1.9)	0.096	147.1(143.5–151.6)	1.3(0.9–1.9)	0.112	148.1(144.0–152.1)
	**IIa**	2.4(1.7–3.2)	< .001	137.9(132.3–143.4)	2.4(1.8–3.3)	< .001	136.3(130.5–142.2)
**NCC**	**IIb**	3.1(2.3–4.1)	< .001	129.5(123.3–135.8)	3.2(2.4–4.3)	< .001	128.6(122.7–134.6)
	**IIIa**	5.8(4.4–7.6)	< .001	112.0(103.1–120.8)	5.8(4.4–7.6)	< .001	111.7(103.0–120.3)
	**IIIb**	7.6(5.7–10.0)	< .001	100.4(90.4–110.4)	10.2(7.9–13.3)	< .001	87.9(77.8–98.0)
	**IIIc**	13.5(10.4–17.4)	< .001	73.0(63.1–83.0)	13.3(10.1–17.7)	< .001	73.5(61.7–85.3)
	**Ia**	1		101.9 (98.6–105.2)	1	-	101.9 (98.6–105.2)
	**Ib**	1.3 (0.5–3.8)	0.613	87.1 (79.7–94.4)	1.3 (0.5–3.8)	0.62	87.1 (79.7–94.4)
	**IIa**	1.0 (0.3–2.8)	0.942	102.9 (96.3–109.6)	1.0 (0.3–2.8)	0.94	103.0 (96.5–109.6)
**TU**	**IIb**	1.2 (0.4–3.4)	0.778	97.5 (90.1–105.0)	2.0 (0.9–4.5)	0.088	92.6 (84.4–100.9)
	**IIIa**	4.3 (1.8–10.1)	0.001	80.9 (67.1–94.7)	3.8 (1.6–8.9)	0.002	79.2 (66.4–92.0)
	**IIIb**	5.4 (2.6–11.3)	< .001	70.7 (58.3–83.1)	5.2 (2.4–11.0)	< .001	72.1 (59.8–84.5)
	**IIIc**	7.4 (3.7–14.8)	< .001	61.6 (47.7–75.5)	9.0 (4.3–19.1)	< .001	55.3 (38.5–72.0)

NCC, National Cancer Center Hospital in Japan; TU, Tokyo University Hospital in Japan

The HRs of each TNM substage (Ib, IIa, IIb, IIIa, IIIb, and IIIc), which were compared to Ia, were 2.1, 2.3, 4.5, 5.5, 9.3, and 19.3 in the current TNM 7^th^ edition system and 1.9, 2.9, 4.1, 6.4, 11.0, and 20.2 in the new TNM staging system, respectively. In the validation sets, the distributions of the HRs of each substage were similar between the new TNM stage and the TNM 7^th^ edition.

**Fig 3:**
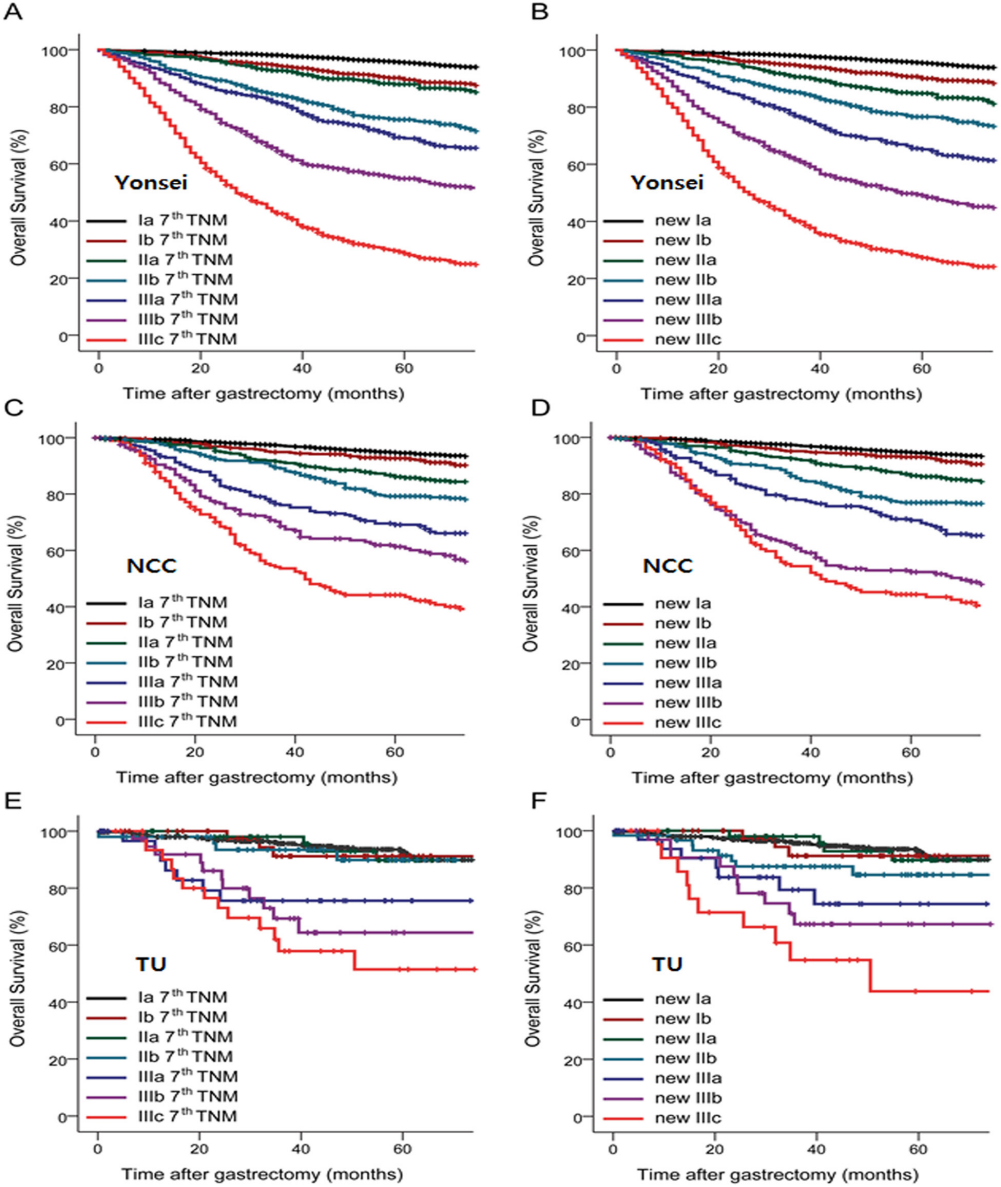
Kaplan-Meier curves for overall survival of each staging system. A) TNM 7^th^ edition system with substages in Yonsei set, B) new TNM staging system with substages in Yonsei set, C) TNM 7^th^ edition system with substages in NCC set, D) new TNM staging system with substages in NCC set, E) TNM 7^th^ edition system with substages in TU set, F) new TNM staging system with substages in TU set. NCC, National Cancer Center in Japan; TU, Tokyo University Hospital in Japan.

According to the Kaplan-Meier curves, prognoses were well-stratified for each substage, not only in the original set but also in the validation sets (Fig 3A to 3F). Harrell’s C-index, which indicates prognostic performance, was 0.799 (95% CI: 0.792–0.806) and 0.799 (95% CI: 0.792–0.806) in the TNM 7^th^ edition and new TNM staging systems, respectively, and there was no significant difference between the two (*p* > 0.999; Table 5). In addition, the C-index of the new TNM staging system was comparable to that of the TNM 7^th^ edition for advanced GC and LN-positive GC (0.726 and 0.703 for TNM 7^th^ edition *vs*. 0.727 and 0.703 for new TNM staging system; *p* = 0.937 and 0.999, respectively). The results from NCC and TU validated the prognostic performance of the new TNM staging system and were nearly identical to the current TNM 7^th^ edition system.

**Table 5:** The prognostic performance of each staging system (Harrell C-index) at each center.

	TNM	New TNM	*p*-value
Overall			
Yonsei	0.799(0.792–0.806)	0.799(0.792–0.806)	>.9999
NCC	0.747(0.736–0.758)	0.748(0.737–0.759)	0.9487
TU	0.703(0.669–0.737)	0.701(0.667–0.735)	0.9668
AGC			
Yonsei	0.726(0.717–0.735)	0.727(0.718–0.736)	0.9374
NCC	0.695(0.680–0.710)	0.700(0.685–0.715)	0.8137
TU	0.702(0.656–0.748)	0.698(0.652–0.744)	0.9510
LN positive			
Yonsei	0.703(0.694–0.712)	0.703(0.694–0.712)	>.9999
NCC	0.696(0.680–0.712)	0.701(0.685–0.717)	0.8251
TU	0.688(0.639–0.737)	0.682(0.634–0.730)	0.9303

NCC, National Cancer Center Hospital in Japan; TU, Tokyo University Hospital in Japan

## Discussion

The present results suggest that the more advanced new N stages were indicative of a worse prognosis, even for the same current pN stage, implying that the anatomical extent of lymph node metastasis is an important factor for gastric cancer prognosis and needs to be applied to gastric cancer staging systems. Accordingly, this new staging system, which reflects the anatomical extent of lymph node metastasis, showed prognostic performance equal to that of the current TNM staging system; moreover, these findings were validated by data from other centers in Japan, supporting this newly proposed staging system as a good alternative to the current TNM staging system.

The most attractive point of our new stating system is its simplicity. With the new staging system, the N stage depends only on the presence of metastatic LNs in each group (LC, GC, and EP), regardless of the number of metastatic LNs. Counting the numbers of both retrieved and metastatic LNs is affected by pathologists or pathology technicians who discover and manipulate LNs in the resected specimens [21], which is one factor related to stage migration. Although this problem can also affect the new staging system, we expect that this new system could reduce any variations or errors in reporting and decrease stage migration, as counting all of the LNs is not necessary. For example, if there is lymph node metastasis in each of the three groups (LC, GC, and EP), it would be sufficient to access only three lymph nodes, one from each group, rather than performing a whole lymphadenectomy. Furthermore, the new staging system unifies preoperative and postoperative staging reports in gastric cancer. As it is difficult to measure the presence and number of metastatic LNs preoperatively with current radiologic tools [4,22,23], an anatomical-based staging system would be more appropriate than a numeric N staging system, which requires counting all suspicious metastatic LNs preoperatively. Additionally, one suspicious metastatic LN in a preoperative image could in fact be more than one metastatic LN. Accordingly, our staging system is not influenced by the number of metastatic LNs and is only affected by the presence of a metastatic LN in at least one of three groups (LC, GC, or EP). Thus, we expect that our newly proposed staging system would make preoperative staging easier, particularly when there is an LN large enough to be detected, and this would also mean that histological results would be those of the preoperative staging.

Another advantage of the new staging system is that it can represent the extent of LN dissection. After a long debate about the advantages of D2 LN dissection [24–28], it is currently considered to be the standard operative procedure in Asian countries [6] and is also recommended in Western countries[12,13] for advanced gastric cancer. Nevertheless, the current numeric staging system does not represent the extent of LN dissection in gastric cancer surgery, as D2 LN dissection is based on an anatomical perspective. Although the current TNM system recommends that at least 16 LNs should be evaluated pathologically [29], this number of LNs does not guarantee a D2 LN dissection, as D1 LN dissection can occasionally be achieved for more than 16 LNs and vice versa. By applying the new staging system, the extent of LN dissection is automatically reflected in the staging system, as EP LN dissection should be performed when using the new N staging system.

The previous Japanese classification [10], which guided the anatomy-based treatment of gastric cancer, provided direct surgical guidance [4] and the anatomic extent of disease necessary to carefully assess gastric cancer [30]. In order to collect information on the anatomical extent of LN involvement, each LN station should be identified and labeled before histopathological assessment. This procedure is typically performed after operation, as the *en bloc* removal of lymph nodes is the most important oncological principle to be maintained during surgery. For this reason, the identification and classification of LN stations could be subjective to arbitrary criteria depending on individual surgeons or pathologists. Therefore, the precise information on LN status might be ambiguous, in particular for LN 7, 8a, 9, and 11p, and only imperfect information may be available despite an increased level of complexity. With this possible reason among others, the Japanese classification system now uses a numeric-based N staging system that is divided into classification [15] and treatment guidelines [6]. Our new N classification is based on the modified anatomical location of metastatic LNs, which can represent the anatomical extent of the disease. This new system could decrease the variations among surgeons or pathologists while providing essential information on the extent of LN metastasis by identifying the location of each LN group, as LC, GC, and EP classifications are unambiguous. Consequently, it would facilitate accurate communication across institutions worldwide regarding LN staging, as this novel concept is intuitive and easier to understand than the original Japanese classification.

The main limitation of this study was that the present findings were not validated in a Western population; rather, the outcomes were only from centers in East Asia. To generalize these results, validation from Western countries is essential, as not only the epidemiology and treatment strategies but also the biology of gastric cancer in Asian patients and Europeans are different [31–33]. Validation in a western population may give further insight into the potential value of the new N staging system and perhaps allow global consolidation of LN staging in gastric cancer.

Several alternative staging systems for estimating the extent of LN metastasis in gastric cancer have been proposed, such as the LN ratio [34–38] (number of metastatic LNs / number of retrieved LNs) and the Kiel classification [39], which attempts to reflect biological tumor properties. However, these proposed staging systems cannot actually solve the aforementioned problems with the current TNM system, as they use the same numeric-based N staging. Moreover, the LN ratio system was criticized for the arbitrarily determined cutoff points, and the Kiel classification failed to be validated by recent studies from the same country [40]. Our novel LN staging system is intuitive and simple, can provide improved communication across institutions worldwide, can be used in preoperative staging, and can represent the extent of LN dissection and the anatomical extent of the disease. Furthermore, our results demonstrate that it offers substantial prognostic performance with validation. This novel N staging system may be a useful alternative to current numeric-based staging systems for gastric cancer.

## Conclusion

Our novel N classification system for gastric cancer, which is based on modified anatomical location, is simple, intuitive, reasonable, easy to apply in clinical practice, and could improve communication among gastric cancer teams and centers. Application of our system may resolve the inherent problems of current numeric N staging systems. Accordingly, we suggest that our staging system may be a good alternative for gastric cancer staging.

## Supporting Information

S1 File Patients’ clinical dataset. (XLSX)

## References

[1] Ferlay J, Soerjomataram I, Dikshit R, Eser S, Mathers C, Rebelo M, et al Cancer incidence and mortality worldwide: sources, methods and major patterns in GLOBOCAN 2012. Int J Cancer. 2015;136: E359–386. 10.1002/ijc.29210 25220842

[2] Jung KW, Won YJ, Kong HJ, Oh CM, Cho H, Lee DH, et al Cancer statistics in Korea: incidence, mortality, survival, and prevalence in 2012. Cancer Res Treat. 2015;47: 127–141. 10.4143/crt.2015.060 25761484 PMC4398120

[3] Jung KW, Won YJ, Oh CM, Kong HJ, Cho H, Lee DH, et al Prediction of cancer incidence and mortality in Korea, 2015. Cancer Res Treat. 2015;47: 142–148. 10.4143/crt.2015.066 25779360 PMC4398104

[4] Sayegh ME, Sano T, Dexter S, Katai H, Fukagawa T, Sasako M. TNM and Japanese staging systems for gastric cancer: how do they coexist? Gastric Cancer. 2004;7: 140–148. 15449201 10.1007/s10120-004-0282-7

[5] Sobin L, Wittekind C. TNM Classification of Malignant Tumours. 5th ed. New York: John Wiley 1997.

[6] Japanese Gastric Cancer A. Japanese gastric cancer treatment guidelines 2010 (ver. 3). Gastric Cancer. 2011;14: 113–123. 10.1007/s10120-011-0042-4 21573742

[7] Fujii K, Isozaki H, Okajima K, Nomura E, Niki M, Sako S, et al Clinical evaluation of lymph node metastasis in gastric cancer defined by the fifth edition of the TNM classification in comparison with the Japanese system. Br J Surg. 1999;86: 685–689. 10361195 10.1046/j.1365-2168.1999.01115.x

[8] Ichikura T, Tomimatsu S, Uefuji K, Kimura M, Uchida T, Morita D, et al Evaluation of the New American Joint Committee on Cancer/International Union against cancer classification of lymph node metastasis from gastric carcinoma in comparison with the Japanese classification. Cancer. 1999;86: 553–558. 10440681 10.1002/(sici)1097-0142(19990815)86:4<553::aid-cncr2>3.0.co;2-d

[9] Hayashi H, Ochiai T, Suzuki T, Shimada H, Hori S, Takeda A, et al Superiority of a new UICC-TNM staging system for gastric carcinoma. Surgery. 2000;127: 129–135. 10686976 10.1067/msy.2000.102171

[10] Japanese Gastric Cancer A. Japanese Classification of Gastric Carcinoma - 2nd English Edition. Gastric Cancer. 1998;1: 10–24. 11957040 10.1007/s101209800016

[11] Nakagawa M, Choi YY, An JY, Chung H, Seo SH, Shin HB, et al Difficulty of predicting the presence of lymph node metastases in patients with clinical early stage gastric cancer: a case control study. BMC Cancer. 2015;15: 943. 10.1186/s12885-015-1940-3 PMC4665830 26625983

[12] Okines A, Verheij M, Allum W, Cunningham D, Cervantes A, Group EGW. Gastric cancer: ESMO Clinical Practice Guidelines for diagnosis, treatment and follow-up. Ann Oncol. 2010;21 Suppl 5: v50–54. 10.1093/annonc/mdq164 20555102

[13] Ajani JA, Barthel JS, Bekaii-Saab T, Bentrem DJ, D'Amico TA, Das P, et al Gastric cancer. J Natl Compr Canc Netw. 2010;8: 378–409. 20410333 10.6004/jnccn.2010.0030

[14] Biondi A, Hyung WJ. Seventh edition of TNM classification for gastric cancer. J Clin Oncol. 2011;29: 4338–4339; author reply 4340–4332. 10.1200/JCO.2011.36.9900 22010017

[15] Japanese Gastric Cancer A. Japanese classification of gastric carcinoma: 3rd English edition. Gastric Cancer. 2011;14: 101–112. 10.1007/s10120-011-0041-5 21573743

[16] Wittekind C, Meyer H-J. TNM Classification of Malignant Tumours (ed 7). Weinheim, Germany, Wiley, 2010.

[17] Harrell FE Jr, Lee KL, Califf RM, Pryor DB, Rosati RA. Regression modelling strategies for improved prognostic prediction. Stat Med. 1984;3: 143–152. 6463451 10.1002/sim.4780030207

[18] Zhang J, Niu Z, Zhou Y, Cao S. A Comparison Between the Seventh and Sixth Editions of the American Joint Committee on Cancer/International Union Against Classification of Gastric Cancer. Ann Surg. 2012 2012/10/13. 10.1097/SLA.0b013e31825eff3f 23059507

[19] Yoon HM, Ryu KW, Nam BH, Cho SJ, Park SR, Lee JY, et al Is the new seventh AJCC/UICC staging system appropriate for patients with gastric cancer? J Am Coll Surg. 2012;214: 88–96. 10.1016/j.jamcollsurg.2011.09.018 22036661

[20] Jung H, Lee HH, Song KY, Jeon HM, Park CH. Validation of the seventh edition of the american joint committee on cancer TNM staging system for gastric cancer. Cancer. 2011 2011/01/13. 10.1002/cncr.25778 24048784

[21] Coburn NG, Swallow CJ, Kiss A, Law C. Significant regional variation in adequacy of lymph node assessment and survival in gastric cancer. Cancer. 2006;107: 2143–2151. 17001662 10.1002/cncr.22229

[22] Kwee RM, Kwee TC. Imaging in assessing lymph node status in gastric cancer. Gastric Cancer. 2009;12: 6–22. 10.1007/s10120-008-0492-5 19390927

[23] Seevaratnam R, Cardoso R, McGregor C, Lourenco L, Mahar A, Sutradhar R, et al How useful is preoperative imaging for tumor, node, metastasis (TNM) staging of gastric cancer? A meta-analysis. Gastric Cancer. 2012;15 Suppl 1: S3–18. 21837458 10.1007/s10120-011-0069-6

[24] Robertson CS, Chung SC, Woods SD, Griffin SM, Raimes SA, Lau JT, et al A prospective randomized trial comparing R1 subtotal gastrectomy with R3 total gastrectomy for antral cancer. Ann Surg. 1994;220: 176–182. 8053740 10.1097/00000658-199408000-00009 PMC1234357

[25] Cuschieri A, Weeden S, Fielding J, Bancewicz J, Craven J, Joypaul V, et al Patient survival after D1 and D2 resections for gastric cancer: long-term results of the MRC randomized surgical trial. Surgical Co-operative Group. Br J Cancer. 1999;79: 1522–1530. 10188901 10.1038/sj.bjc.6690243 PMC2362742

[26] Memon MA, Subramanya MS, Khan S, Hossain MB, Osland E, Memon B. Meta-analysis of D1 versus D2 gastrectomy for gastric adenocarcinoma. Ann Surg. 2011;253: 900–911. 10.1097/SLA.0b013e318212bff6 21394009

[27] Seevaratnam R, Bocicariu A, Cardoso R, Mahar A, Kiss A, Helyer L, et al A meta-analysis of D1 versus D2 lymph node dissection. Gastric Cancer. 2012;15 Suppl 1: S60–69. 22138927 10.1007/s10120-011-0110-9

[28] Songun I, Putter H, Kranenbarg EM, Sasako M, van de Velde CJ. Surgical treatment of gastric cancer: 15-year follow-up results of the randomised nationwide Dutch D1D2 trial. Lancet Oncol. 2010;11: 439–449. 10.1016/S1470-2045(10)70070-X 20409751

[29] AJCC Cancer Staging Manual. New York: Springer; 2010.

[30] Washington K. 7th edition of the AJCC cancer staging manual: stomach. Ann Surg Oncol. 2010;17: 3077–3079. 10.1245/s10434-010-1362-z 20882416

[31] Theuer CP, Campbell BS, Peel DJ, Lin F, Carpenter P, Ziogas A, et al Microsatellite instability in Japanese vs European American patients with gastric cancer. Arch Surg. 2002;137: 960–965; discussion 965–966. 12146999 10.1001/archsurg.137.8.960

[32] Naylor GM, Gotoda T, Dixon M, Shimoda T, Gatta L, Owen R, et al Why does Japan have a high incidence of gastric cancer? Comparison of gastritis between UK and Japanese patients. Gut. 2006;55: 1545–1552. 16603635 10.1136/gut.2005.080358 PMC1860129

[33] Yamaoka Y, Kato M, Asaka M. Geographic differences in gastric cancer incidence can be explained by differences between Helicobacter pylori strains. Intern Med. 2008;47: 1077–1083. 18552463 10.2169/internalmedicine.47.0975 PMC3732488

[34] Yu W, Choi GS, Whang I, Suh IS. Comparison of five systems for staging lymph node metastasis in gastric cancer. Br J Surg. 1997;84: 1305–1309. 9313721

[35] Marchet A, Mocellin S, Ambrosi A, Morgagni P, Garcea D, Marrelli D, et al The ratio between metastatic and examined lymph nodes (N ratio) is an independent prognostic factor in gastric cancer regardless of the type of lymphadenectomy: results from an Italian multicentric study in 1853 patients. Ann Surg. 2007;245: 543–552. 17414602 10.1097/01.sla.0000250423.43436.e1 PMC1877031

[36] Kim CY, Yang DH. Adjustment of N stages of gastric cancer by the ratio between the metastatic and examined lymph nodes. Ann Surg Oncol. 2009;16: 1868–1874. 10.1245/s10434-009-0430-8 19434459

[37] Latengbaolide A, Lin D, Li Y, Xu H, Chen J, Wang B, et al Lymph Node Ratio Is an Independent Prognostic Factor in Gastric Cancer After Curative Resection (R0) Regardless of the Examined Number of Lymph Nodes. Am J Clin Oncol. 2012 2012/05/02. 10.1097/COC.0b013e318246b4e9 22547011

[38] Cheong JH, Hyung WJ, Shen JG, Song C, Kim J, Choi SH, et al The N ratio predicts recurrence and poor prognosis in patients with node-positive early gastric cancer. Ann Surg Oncol. 2006;13: 377–385. 16450215 10.1245/ASO.2006.04.018

[39] Warneke VS, Behrens HM, Hartmann JT, Held H, Becker T, Schwarz NT, et al Cohort study based on the seventh edition of the TNM classification for gastric cancer: proposal of a new staging system. J Clin Oncol. 2011;29: 2364–2371. 10.1200/JCO.2010.34.4358 21537040

[40] Reim D, Loos M, Vogl F, Novotny A, Schuster T, Langer R, et al Prognostic Implications of the Seventh Edition of the International Union Against Cancer Classification for Patients With Gastric Cancer: The Western Experience of Patients Treated in a Single-Center European Institution. J Clin Oncol. 2012 2012/12/06. 10.1200/JCO.2012.44.4315 23213098

